# Reflections on the challenges of understanding racial, cultural and sexual differences in couple relationship research

**DOI:** 10.1111/1467-6427.12044

**Published:** 2014-05-08

**Authors:** Jacqui Gabb, Reenee Singh

**Keywords:** qualitative research, systemic psychotherapy, reflexivity, racial and cultural differences, sexuality, couple relationships

## Abstract

In the field of systemic psychotherapy there has been much recent interest in the areas of culture and reflexivity, and in working with couples. In this article we reflect on the process of conducting research in these areas. Drawing on findings from a large, national, empirical mixed-methods study on long-term relationships, we use two examples from the data to illustrate the complexity of researching across racial, cultural and sexual differences, in terms of research design and sampling, fieldwork and research practice, and making sense of multidimensional data. We point to findings that suggest that notions of coupledom are culturally constructed and thus challenge straightforward ideas of the procreative, sexually active couple dyad, separate from intergenerational extended families. The clinical significance of the findings for both lesbian, gay, bisexual or queer and culturally diverse couples and families are discussed.

**Practitioner points**

- Cultural or racial matching is not a sufficient condition for engagement and empathy with couples and families.
- Critical reflexivity about similarity and difference is essential in cross-cultural systemic practice.
- ‘The couple’ and its distance from the extended family may be defined differently in different cultures.
- One research tool used in this project, the emotion map, appears to have utility in clinical practice with couples and families.

## Introduction

The idea of reflexivity is central to both psycho-social qualitative research and cross-cultural systemic practice. In her recent book Krause (2012) traces the history of reflexivity in systemic psychotherapy and the place of reflexivity in cross-cultural clinical work. When working across culture, professionals run the risk of assuming that their taken for granted ideas of selfhood and kinship are universal (Krause, 1998; Singh, 2009). Cross-cultural research may provide the greatest opportunities for reflexivity, with each difference and similarity between us and our research participants inviting us to reflect on our connections and departures from what is known to us, from our own cultural vantage points. Taking account of sexuality (same-sex, bisexual and queer) and its context in or opposition to dominant heteronormative understandings of intimate life and loving relationships similarly requires the researcher and clinician alike to take stock, think again and question taken for granted assumptions.

Where does this journey begin? For us, the authors of this article, it began in deciding to work together, because of or despite our differences with regard to race, culture and sexual orientation, and the different research–professional practice positions that we occupy. The occasion was a research project called ‘Enduring love? Couple relationships in the 21^st^ century’, completed over a 2-year period (2011–2013) and funded by the Economic and Social Research Council (RES-062–23–3056). This psycho-social study aimed to interrogate what helps couples to stay together and how they work to sustain their relationships. Alongside a large scale online survey (*N* = 5445), 50 couples were interviewed, using a range of qualitative methods that were designed to explore their everyday lived experience. The qualitative sample consisted of white UK (*n* = 76) and black and minority ethnic individuals (BME) (*n* = 24); two-thirds of participants self-identified as heterosexual (*n* = 66) and one-third as lesbian, gay, bisexual or queer (LGBQ) (*n =* 34). The research team comprised two principal investigators and several consultant researchers. The diverse composition of our research team was intentional, aiming to facilitate critical attention to couple diversity at all stages of the research process, including the experience of ‘race’, culture, class, generation and sexual orientation.

Throughout the course of our work together, we were reminded that similarity and difference between researcher and participant are constructed afresh in each research encounter, with different aspects of difference (and similarity) coming to the fore on each occasion. This highlighted how ideas of researcher–participant matching, however well-intentioned, are inevitably problematic as they rely on assumptions of homogeneity in sample groups. A striking point that emerged from our data and research practice was how couple diversity within particular racial, cultural or sexual groups challenged taken for granted constructs of coupledom.

In this article we reflect on how differences and similarities are understood (in research and clinical practice) and the role of the researcher or clinician in racialized contexts. We begin by introducing the research study; its aims, questions, methodology and design. We review the literature on racial and ethnic matching in research and the difficulties of defining the term Asian. We draw on the findings from the study to illustrate how we understood and used researcher–participant racial, cultural and sexual differences, as well as the differences in our findings. Thus, the article is aimed at both systemic psychotherapists and family and couple researchers. We conclude by outlining the clinical implications of our research experience and findings.

## Enduring love? Couple relationships in the 21^st^ century

Enduring love? was an empirical investigation that examined the ways in which gender, generation and parenthood get inscribed in meanings and practices around the idea of the couple. Much recent policy, academic and professional research has focused on the stressors that can contribute to relationship breakdown (Walker *et al*., 2010) and the adverse impact of marital distress and family fragmentation on the health and wellbeing of men, women and children (Markham and Halford, 2005). Our research was designed to shift attention onto the things and qualities that help people sustain their relationships. In doing so our aim was to focus attention on relationships that endure – as both enduring relationships of quality and good enough or endured relationships. While findings from our large-scale survey have informed our thinking, in this article we draw on the qualitative data.

Our previous research on family relationships (Gabb, 2008, 2009) and sexuality (Gabb, 2012) has demonstrated the usefulness of a qualitative, mixed-methods (QMM) approach in accessing accounts of emotional life and everyday relationship practices. The qualitative methods that were deployed in this project included diaries, emotion maps, individual interviews and photo elicitation couple interviews. These data were supplemented by and embedded in research diaries and field notes. The first two methods completed by the participants were diaries and emotion maps. Emotion maps are a graphic technique pioneered by co-author Jacqui Gabb in her research on family relationships (Gabb, 2008), generating data on participants' emotional geographies and mundane relating practices, located in the material environment, in this case the couple home (Gabb, 2009). Emotion maps begin with a floor plan sketch of the home, which is transferred to a Word document using Microsoft Draw or another similar program. A copy of this floor plan is then returned to each participant, along with a set of coloured emoticon stickers denoting laughter, being happy or nonplussed, sad, upset, grumpy/angry, and love/affection. Over the course of one week, individuals place an emoticon sticker on their floor plan when different kinds of interactions occur, using different colours to denote different people, such as the participant, their partner, children (if any), family, friends, pets and so on.

Diaries generate temporal data on everyday routines, highlighting the internal ‘couple lexes’ which otherwise remain private (Gabb, 2008). This brings to the fore the repertoires and frameworks of emotional understanding shared by the couple or family group, that is their internal relationship discourse. Clear guidelines are provided on the kinds of experience that might be described including time spent together, apart and with other people, and how or whether these activities inclined them to think about their relationship in some way. The participants are also encouraged to record one good moment and one challenging moment in each day. The length and format of completed diaries ranged from a few scrappy pages to lengthy and beautifully illustrated documents. Both diaries and emotion map data are then used as a device to facilitate talk on lived examples of couple relationships. Individual open interviews were completed to provide biographical information, with each participant responding to the same single question: Tell us about your relationship; how does it work? Couple interviews focused on a series of collages designed to interrogate the management of public–private boundaries (Gabb, 2013) and how cultural meanings and personal experience intersect (Fink, 2008). These data add another dimension to biographically embedded individual interviews (Heaphy and Einarsdottir, 2013). Combined, the interviews produce comparative data on gendered his and hers marriage experience (Hochschild, 1989).

Psycho-social QMM research is designed to produce a situated, richly textured account of where, when, how and why couple relationships are experienced. Developing out of post-war work completed by the Tavistock Institute into the pluralist forms which can be seen today (Walkerdine, 2008), psycho-social studies question what constitutes truth, knowledge and subjectivity, affording attention to the researcher as a constitutive component in what and how we know. Field notes and the researcher diary therefore comprise a crucial dimension in a psycho-social methodology. They provide both a means to explore issues of counter-transference (Hollway and Jefferson, 2000) and the impact of the researcher on the subject or object of study. Rich and personally revealing field notes are not separated from analysis or restricted to research context: researcher subjectivity is interrogated as a rich source of evidence (Thomson *et al*., 2012). The research relationship is recognized as a dynamic site where roles and interactions shift between the various participants.

The QMM approach that we deployed elicited a wealth of rich and multidimensional data; however, degrees of candidness inevitably differed from one couple to the next and between one individual and another. At times some accounts appeared packaged and rehearsed, perhaps indicating an emotional defence or sense of personal caution; others were disarmingly open. Levels of disclosure were often personal to the participant but at other times gendered, generational and cultural factors appeared to shape what could and could not be spoken. This was often the case on the subject of sex and intimacy; something that we will address in our discussion. Most participants stated that it was rewarding to take part in the study; indeed, many asserted that they had gained insight into themselves or their relationship through the research process. Some perceived it as akin to couple counselling and, as such, participation served as a positive point of intervention. At no point, however, did the researchers volunteer advice or make a directive intervention in the couple relationship.

### Sampling strategies: mixing and matching

The qualitative research sample comprised women and men from three age cohorts (18–34, 35–54 and 55–65+), with equal numbers of parents and childfree couples. Purposive sampling ensured social diversity, including education or socioeconomic status, sexuality, race or ethnicity and religious belief. Our research strategy built on the premise that insider knowledge would both facilitate our access to these hard to reach populations and also sensitize the research project to the specificities of BME groups and non-heterosexual participants. So, for example, research team expertise in the field of Asian couple relationship support ensured that our research methods were attuned to cultural differences and sensitivities in this population. Questions in the survey were thus formulated in ways that extended beyond Western couple experiences. The collages were constituted to enhance representations of diversity and take on board cultural sensitivities around the acknowledgement and display of intimacy and sex. In this article, in particular, we want to speak to the issues raised in researching across racial and ethnic differences and being attentive to the ethical, emotional, analytical and methodological dilemmas that are generated by racial subjectivities (Twine, 2000).

Feminist and sexuality studies have convincingly argued the merits of commonality between interviewee and interviewer (for example Maynard and Purvis, 1994). A researcher's insider status can enhance research in many ways, literally and conceptually opening doors (Gabb, 2004) and affording an epistemic privilege (Fuss, 1991) that facilitates both rapport and the potential for greater insight on the topic of investigation. The Enduring love? project was attuned to issues of insider–outsider dualism; in particular, how boundaries between the researcher and the interviewee remain in flux in terms of recognition and identification. It was, therefore, with some degree of trepidation that we proceeded down the route of what has been formerly called racial matching. Drawing on the involvement and expertise of one of the project strategy group partners, the Asian Family Counselling Service, the project principal investigators (co-author Gabb and colleague Janet Fink) decided to include an Asian subset in our wider sample cohort. Reenee Singh (co-author), a researcher and trained systemic psychotherapist, joined the research team, taking the lead on recruitment and fieldwork in this dimension of the sample. The pragmatics of the research process, the slipperiness of racial identifications and the particular research interests of Singh, however, combined to unravel this idea of a relatively closely defined sample subset.

Ideas of racial matching emerged in sociological research in the context of the 1960s civil rights movement in the USA through a desire to better understand and appreciate black experience (Rhodes, 1994). Notwithstanding these worthy intentions, ascribing insider status to a researcher on the basis of racial identity raises many unsettling questions. Race or colour matching is rooted in a realist epistemology (Phoenix, 1994). Age, class, education, sexuality, national origins, religious belief and so forth are equally important signifiers in the matching of researcher and research. Race is not simply embodied, through, for example, identity category, cultural origin, or lifestyle; neither is it straightforwardly at the behest of the research project or researcher to privilege one marker of experience or self-identity above another. In a critical reflection on mixed-race methodologies, Minelle Mahatni poses two important questions that tease out some of the taken for granted assumptions around sameness and difference. She asks: ‘What constitutes an insider position in critical mixed-race research?’ and how do the researcher's personal ‘subconscious desires and experiences influence the stories we feel compelled to tell in critical mixed-race theory?’ (Mahtani, 2012, p. 157). Consideration of these two questions has proved to be central to the sampling strategies deployed in the Enduring love*?* project and our reflexive approach more generally in researching interracial relationships.

Feminist epistemological thinking recognizes that all knowledge is partial and situated (Collins, 1990). We embark upon research with ‘maps of consciousness’ (Haraway, 1991, p. 191) that are influenced by our own gender, class, national and racial attributes. The insights that we advance and the knowledge offered on our subject, remain, therefore, always partial because of our positionality or situatedness. The dynamic blend of our race, class, gender, nationality, sexuality and other formative and constitutive identifiers inform all aspects of the research. Moreover, racial differences and connections are neither stable nor immutable. Thus, it may be apposite to think of shared spaces in research rather than shared racial identities or matched belonging (Mullings, 1999). Critical mixed-race theory has thus made compelling arguments around the need to problematize and look reflexively at the complex identities and preconceptions that we bring to the field. Studies of interracial relationships require similar critical attention.

In this article, in the sections below, we speak to the dualities that shape research in the field, exploring how ideas of absence and presence, sameness and difference, and insider and outsider status inform and inflect the research process, the data generated and our collective analysis of these data. Who we are, as researchers and as women inside and outside the academy, remains crucial. Advancing a psycho-social approach, we simultaneously acknowledge the situatedness of the researcher alongside the ways that the emotional, psychic and lived biography of the researcher is embedded in the research at every level.

### Defining Asian

As an Asian systemic psychotherapist and researcher, Singh has a particular interest in how culture shapes the construction of couplehood. From her personal experiences of growing up in India and from her clinical practice, she has the view that Asian couples are embedded in extended families (Nath and Craig, 1999). In non-Western family forms, the primary dyad is not always the couple: it could comprise the father–son or mother–son, or a dyad based on affinal rather than biological ties (Falicov, 1995; Krause, 1998). The research project thus presented Singh, and the project team more widely, with an opportunity to explore the ways that the Asian couple experience may differ from its English counterparts, and to develop more nuanced understandings of differences among the inevitably diverse range of couples who could be included in the sample and in this sampling category in particular.

As a first step in identifying couples to interview, demographic information provided in the quantitative survey data was used to identify participants who described themselves as Asian. Gunaratnam (2003) points to the risk of flattening out complexity and minimizing intra-group difference in research, as this has the potential to reproduce wider forms of essentialism, stereotyping and racism. With hindsight, therefore, the sampling category could have been further refined, for example, as ‘South Asian’, although this is in itself a homogenizing term. Combined logistical considerations and participant interest identified five couples from the survey to participate in the in-depth qualitative study. This subset of Asian couples turned out to be a profoundly disparate group. Of this group, two individuals identified as ‘mixed race’ (one with Filipino heritage and one Hong Kong Chinese); one had migrated from India, one from Taiwan and one was South Asian Muslim. Quite unexpectedly, these individual participants were all in relationships with White British partners; hence all five couples were in interracial relationships. A further two couples were recruited to this subset through snowballing in Singh's personal social networks. Of these two couples, one was an elderly South Asian (Guajarati) couple and the other a lesbian couple in which both partners identified as having English and Indian heritage. The heterogeneity of the couples presents rich opportunities to think about diversity. In the sections that follow we explore the personal reflections of Singh as presented in her research diary, and, as part of the psycho-social methodology, our critical interrogation of these and other participant data:

As a migrant from India, married to a White English man, perhaps I can relate most to the experiences of the two migrant women in my sample. One of my clinical interests is in the area of intercultural relationships and through my experiences of interviewing; I have become increasingly interested in the dynamics of intercultural relationships. However I feel quite strangely ‘other’ to the same-sex couple mixed-race couple …

I re-read Usha and Leah's diaries today and felt quite moved. There is something so innocent about their relationship although even while I am saying this, I realize that I run the risk of exoticising the ‘other’. The passages about who is going to bear the first child and the talk of fertility treatment … it made me realize how much more planned families with same-sex couples have to be … I guess my predominant thought following my experience is about how different lesbian relationships are from heterosexual relationships in the range of gendered roles available. Although there did seem to be power differences, their relationship seemed far more equitable than that of many heterosexual couples. (Singh: field notes)

Usha and Leah both have higher educational qualifications; they co-habit and are in the younger and mid-range age cohorts. There are, therefore, several points of ready identification between researcher and participant – age, class and perceptions of parenthood as part of the long-term couple narrative. Differences in sexual orientation, however, obscure such similarities. Singh's perceived otherness to this lesbian couple stands in stark contrast to that of co-author Gabb who has both previously studied lesbian parenthood and also identifies as lesbian and the birth mother of an adult son. For her, the diaries of Usha and Leah were both familiar and affirming. In this context, her insider status afforded a notably different perspective on the diary data to that of Singh. The crafting of family narratives was taken for granted and she questioned what lived material differences there were in the everyday experience of same-sex and heterosexual couples. Her personal experience and academic research has called into question embodied readings of gender and power in same-sex couple relationships (Gabb, 2005) and how these are deployed to establish categorical differences (Gabb, 2011). The sameness and differences of Singh and Gabb in this context bring to the fore, therefore, how difference can serve to obscure similarities and conversely how similarities serve to obscure difference. The example also illustrates how troublesome it is to read and research experience through ideas of matching.

### Where is the couple relationship?

**Figure 1:**
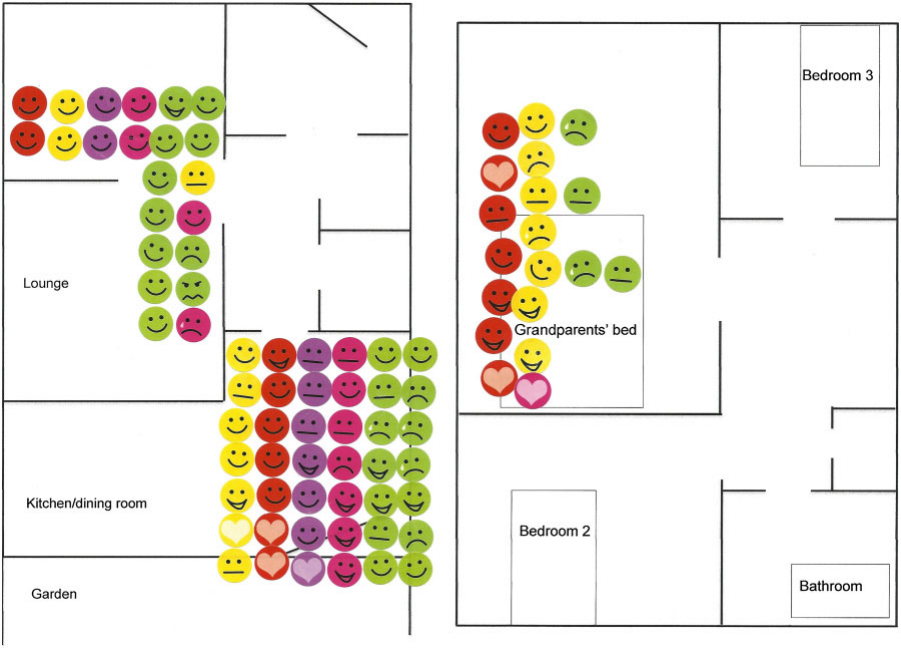
Anuradha's emotion map. The original colours used were red, Anuradha; yellow, Prakash; green, granddaughters; purple, son; pink, daughter-in-law. The coloured figure can be found in the online version of this article.

Another area where our situatedness became crucial in making sense of the data was in our assumptions and understandings of what constitutes a couple relationship. This raises important questions about the feasibility of researching couple experience across cultural differences and the problematic of prevailing Western cultural constructions of the couple relationship. Across the dataset, most of the emotion maps depicted the couple alone at least on some occasions. When these data included other people, during interviews the participants would typically explain the circumstances – for example, a friend or relative had been visiting; and the lives and living arrangements of parents and children are inevitably intertwined. Confirming previous research findings that emphasize the boundaries set up around public and private space (Gabb, 2008), the one place where the couple was distinctly located was the martial bedroom, although a child or a pet might snuggle up with them in bed. However, the emotion maps of Prakash and Anuradha reveal a quite different picture of couple experience. This South Asian (Guajarati) couple, aged in their 60s, live with one of their sons, a daughter-in-law and their two granddaughters (aged 6 and 3). Anuradha and Prakash's emotion maps depict all six of them eating together and both the grandchildren sharing their bed. (See Figure 1)

In this cultural context, in the patriarchal extended family it is expected that adult sons will live with their aging parents. Women marry into the husband's family and the grandmother takes an active role in running the household and looking after the children. Anuradha explained how her own mother-in-law had looked after her children when they were young and now it was simply her turn. They hardly spoke about their couple relationship in their individual interviews. Anuradha's primary relationship seemed to be with her grandchildren; that of Prakash, with God. In the couple collage interview, when asked about celebrations, their descriptions of their birthdays and other celebrations always included the children, grandchildren and other relatives. When asked if they ever spent time alone together, they said that they sometimes went to the supermarket together, just the two of them. They did not appear to comprehend or feel the need for private couple time. In response to the collage that depicted images of close embodied affection and sexual intimacy, Anuradha looked shocked and said: ‘We are not doing these things’.

Anuradha and Prakash's family form was to some large degree familiar to that of Singh, replicating her cultural background; however, it also felt profoundly different, as illustrated in this extract from her research diary:

Anuradha and Prakash are such a lovely couple but I did feel out of joint today with them. I guess that must be one of the problems of working across culture. When one is faced with such profound differences, it is hard not to feel different. I felt like an outsider, looking in. (Singh: research diary)

Looking back on her field notes and in conversations with the research team, Singh became struck by a sense of disjuncture and discomfort – when similarity and difference come together at the same time. Each home in the Asian subset felt distinctly different from the others: each had its own smells, sounds and unique feel. Singh recalled that as soon as she entered Anuradha and Prakash's home she felt transported back to India. Their house reminded her of many of the homes of relatives and friends in India – the same sweet, milky tea (which is politely accepted) and the delicious hot samosas.

However, there are also marked differences between Singh and this couple, notably that of age, or perhaps more accurately stated, generation. Anuradha is in her early 60s and Prakash in his late 60s. Their relationship, formed through an arranged marriage, has lasted for 40 years, nearly equalling Singh's entire lifetime. Her own couple relationship, in contrast, is relatively new. She went against cultural beliefs and expectations in marrying somebody from a different race and culture. She could, therefore, more readily identify with other younger interracial couples in the sample, such as Usha and Leah. These couples all seemed to be grappling with similar issues of how to negotiate language, food and faith differences in their everyday lives and couple relationships.

When talking to Anuradha and Prakash, Singh became acutely aware of the reliance of this couple on shared beliefs and ideas about, for example, the role of extended family and grand-parenting. The *mandir* (temple) is a focal point for their family and community life. Gendered roles appear to be far more clearly defined in their couple relationship than those of Singh and the rest of her research participants. Anuradha has the primary responsibility for cooking and looking after the grandchildren, even though she used to work in the family corner shop. In addition to the generational difference, class and education was another differentiating factor that set researcher and participant apart. Anuradha struggled to keep her diary, as she said her English is not good enough. There were no research funds available for translation, thus, differences in first language shaped both the research encounter and data generated, further cleaving apart these separate worlds. Anuradha and Prakash value education and one of their two sons has a doctoral qualification. Singh was left reminded of how education allows her access to a ‘White world’ in which this older South Asian couple are displaced.

### Worlds apart: clinical implications

The data of Anuradha and Prakash raise important issues for the Enduring love? project and how we research and understand couple relationships more widely. Studies that aim to establish measures of relationship satisfaction and qualitative studies on couple relationship experience both presuppose an intimate dyadic couple as fundamental and foundational. However, as the eponymous name of the Asian Family Counselling Service suggests, this is perhaps both a misguided presumption and also the wrong starting point for inquiry. Cultural constructions of the couple and coupledom are deeply embedded in Western culture; a conjugal pairing that comprises the procreative sexual family (Fineman, 1995). The two couples that we have included here disrupt this couple narrative, in markedly different ways. Others were disruptive of the couple norm in their own individual ways. Usha and Leah unravel naturalizing discourses on how couples conceive children and gendered understandings of couple roles in a relationship. For Anuradha and Prakash, their couple relationship is so steeped in cultural expectations of intergenerational extended family care that contemporary Western understandings of the intimate couple are rendered meaningless.

So, when couple intimacy is displaced, what is it that keeps Anuradha and Prakash together? In what ways is this different from the processes that make for ‘enduring love’ in the other couple relationships? Consistent with the literature (Kakar, 1981; Nath and Craig, 1999), preserving harmonious relationships with in-laws and valuing family relationships would seem to be an important predictor of successful long-term couple relationships in this South Asian context. However, what happens in intercultural – or, for that matter, monocultural – couple relationships, when individuals might come together with very different ideas on whether their couple relationship should be separate from or embedded within the extended family? Among South Asian communities in the UK many young people who have grown up in this country are expected to marry people from their parents' countries of origin. In such cases, partners may have radically different ideas about what constitutes coupledom; one drawing on Western norms and the other on ideas from the parents' non-Western country of origin.

As a pointer for clinical practice, perhaps one of the first tasks when working with such couples is for the clinician to identify their personal ideas and each partner's ideas of what constitutes a couple relationship. Put simply, ‘what is a couple'? And what is the distance or closeness of this couple relationship from the rest of the family? Clinicians cannot assume the universal utility of ideas like dating and couple time. In many cultures sex is for the purposes of procreation. As our research here shows, it cannot be assumed that all couples, especially older couples, should be or want to be sexually active. Conversely, for some couples sex is not and never will be procreative. There are important parallels between researching across cultures and working clinically with couples across cultures. One significant similarity seems to be the assumption that being culturally, racially or sexually matched is a sufficient condition for engagement in the therapeutic or research encounter. Similarity and difference are complex, shifting, intersectional and always relational. Retaining curiosity about and being attentive to similarities and differences is fundamental to both research and clinical practice.

Many LGBQ individuals continue to experience negative or mixed reactions from mental health professionals (Department of Health, 2006); they can feel pathologized by therapists for their sexuality (Evans and Barker, 2010) and heterosexist assumptions may lead to poor understandings of their clinical needs (Galgut, 2005). Research suggests that bisexuality may be a concept and identity that is particularly misunderstood (Klein, 1993). There is, however, increasing awareness of LGBQ relationships and the needs which these individuals may have, with understandings and sensitivities being advanced through wider sociocultural attitudinal tolerance and continuing professional development training (Shaw *et al*., 2009). It is becoming accepted that therapists need to be aware of sexuality issues and also LGBQ couple norms if couple therapy is to be successful (Bepko and Johnson, 2000). There is a shift in emphasis, placing responsibility back onto the counsellor as LGBQ couples assert their unwillingness to educate clinicians; instead, actively seeking out a knowledgeable counsellor (Grove and Blasby, 2009) who is aware of the particular issues that they may face (Bepko and Johnson, 2000).

The literature on cross-cultural family therapy shows that, although cultural and ethnic matching can ease the engagement process and hence improve outcomes (Ito and Maramba, 2002), it can also contribute to clients' worries about confidentiality and lead to therapists' losing curiosity (Singh, 2013). While South Asian children and adolescents in families may be more at risk for mental health difficulties than their White English counterparts, they are far less likely to access child and adolescent mental health services (Raval, 2003; Stein *et al*., 2003). One possible reason why they do not access services is that clinicians may draw on Western notions of the family (Singh, 2009) and the couple and hence unwittingly pathologize or fail to recognize the strengths, resiliencies and resources of the extended families that they meet in their consulting rooms.

In this article we have pointed to some of the ways in which cultural biases such as these may take shape. We suggest that in being attentive to our own situatedness, and how this is generated through and impacts on our perceptions and understandings of the lives of our participants and clients, we can enhance our appreciation of, and thus, our work with couples in all their diversity.

We found that the research methods that included visiting couples at home and asking them to keep research diaries and emotion maps may be a valuable tool that could be adapted to engaging and working with couples and families in systemic clinical practice (see Gabb and Singh, forthcoming). Boyd-Franklin and Hafer-Bry (2000) discusses the importance of home and community visits in engaging with minoritized groups. In this research project, the emotion maps of Anuradha and Prakash yielded rich situated data about their everyday couple practices that may have been missed out on if we had used more conventional research methods such as interviews. This was particularly important in this case, given the couple's difficulties with the English language. Extrapolating from this example, clinicians working with minority ethnic families could use home visits and emotion maps to interrogate lives lived at home that may not translate into the encounter with the couple or family in a consulting room.

In this article we have drawn on the specific subset of participants who were recruited and interviewed by Singh. As it is relatively small in number we do not attempt to generalize from it. In future analysis we plan to broaden this out to include the other Asian, BME and interracial couples included in the project sample, exploring how different researchers experience and interpret similarities and differences which are perceived and experienced. In future research, beyond the scope of the Enduring love? project, it would also be useful to incorporate couples' personal reflections on the interview encounter and how they experience and perceive similarities and differences between participant and client and researcher and clinician.

Burbach and Reibstein (2012) suggest that couple work is synonymous with systemic work. Hence, working with pairs and dyads in families and professional systems is an integral part of our jobs as systemic psychotherapists. What we bring to this work, then, are our own deeply held beliefs, ideas and recognitions of what constitutes a couple and coupledom. We hope that this article highlights the need for clinicians to engage in self-reflexive scrutiny of the assumptions they bring to their clinical work with couples; from a position that neither exaggerates nor denies difference. Burnham (2012) extends his idea of the gender, religion, race, age,(dis)ability, culture, class, education, employment, sexuality, and spirituality (GRRAACCEESS) to that of a ‘collide-scope’, to ‘demonstrate the rich, complex, sometimes random, unpredictable relationship between the different aspects of a person's experience within the complexity of social relations’ (p. 143). The examples from this article demonstrate the need to go beyond a simple matching of race, culture, class and sexual orientation, moving to a position that embraces complexity and difference in order to understand and work systemically with couples.
